# Scientific Items

**Published:** 1878-06-20

**Authors:** 


### *Scientific Items.*

### PUTREFACTION.

Professor Tyndall has experimented with six hundred different test-tubes, containing every variety of infusion, and has found that when the tubes have been heated to boiling, in contact with air, free of dust and germs, and then abandoned to themselves, they have never, in any one case, undergone the action which we style putrefaction, but, on being exposed for two or three days to the open air, the phenomenon of putrefaction at once sets in. The conclusion from these experiments is, that putrefaction and fermentation are due to germs floating in the air, and that there is less danger from nauseous gases in zymotic diseases than from germs likely to be concealed in water. Some form of mote, germ or sporule is necessary to initiate the disease, and these are more readily conveyed by water than by the air.—*Exchange.*

### ARTIFICIAL LARYNX.

Dr. Foulis, of Glasgow, in a patient whose larynx had been extirpated, substituted a metal contrivance which takes the place of the natural organ, and through which the patient is enabled to articulate wonderfully well. The sounds are somewhat monotonous, but the vowels are clear and distinct. What next?

### AN ARMY OF ANTS.

Mr. Belt gives the following graphic account of the excitement caused by a marching column of *ecitons* in the primeval forests of Nicaragua:

"My attention was generally first called to them by the twittering of some small birds belonging to different species. On approaching, a dense body of the ants, three or four yards wide, and so numerous as to blacken the ground, would be seen moving rapidly in one direction, examining every cranny and underneath every fallen leaf. On the flanks, and in advance of the main body, smaller columns would be pushed out. These smaller columns would generally first flush the cockroaches, grasshoppers, and spiders. The pursued insects would rapidly make off, but many, in their confusion and terror, would bound right into the midst of the main body of ants. At first, the grasshopper, when it found itself in the midst of its enemies, would give vigorous leaps, with perhaps two or three of the ants clinging to its legs. Then it would stop a moment to rest, and that moment would be fatal, for the tiny foes would swarm over the prey, and, after a few more ineffectual struggles, it would succumb to its fate, and soon be bitten to pieces and carried off to the rear. The greatest catch of the ants was, however, when they got among some fallen brushwood. The cockroaches, spiders, and other insects, instead of running right away, would ascend the fallen branches and remain there, while the host of ants were occupying all the ground beneath. By and by, up would come some of the ants, following every branch, and driving before them their prey to the ends of the small twigs, where nothing remained for them but to leap, and they would alight in the very throng of their foes, with the result of being certainly caught and pulled to pieces.—*From "Our Six-footed Rivals," in Popular Science Monthly for January.*

### NEW FREEZING PROCESS.

A Californian has invented an ingenious water faucet, through which, if water is drawn, it comes out as cold as ice-water. The faucet contains numerous small tubes inclosed in larger ones, and between the outside of one and the inside of the other certain chemicals are packed, which produce the desired effect.

